# Simultaneous single-cell calcium imaging of neuronal population activity and brain-wide BOLD fMRI

**DOI:** 10.1038/s41592-026-03154-2

**Published:** 2026-07-15

**Authors:** Rik L.E.M. Ubaghs, Roman Boehringer, Markus Marks, Helke K. Hesse, Mehmet Fatih Yanik, Valerio Zerbi, Benjamin F. Grewe

**Affiliations:** 1 Institute of Neuroinformatics, ETH Zurich and University of Zurich, Zurich, Switzerland; 2 Division of Engineering and Applied Science, California Institute of Technology, Pasadena, USA; 3 Hesse - Optical Consulting, Gießen, Germany; 4 Department of Psychiatry, Faculty of Medicine, University of Geneva, Genève, Switzerland; 5 Department of Basic Neurosciences, Faculty of Medicine, University of Geneva, Genève, Switzerland; 6 ETH AI Center, ETH Zurich, Zurich, Switzerland

**Keywords:** Neuro-vascular interactions, Ca2+ imaging, Fluorescence imaging, Functional magnetic resonance imaging

## Abstract

Functional magnetic resonance imaging (fMRI) based on the blood-oxygen-level-dependent (BOLD) signal is widely used to study brain activity non-invasively. However, the relationship between the local neural population activity and vascular activity, as measured by BOLD fMRI, remains incompletely understood. Here we show that simultaneous measurement of cellular calcium (Ca^2+^) activity and whole-brain BOLD fMRI in awake mice can reveal spatially specific relationships between neurons and the surrounding vasculature. We introduce an MRI-compatible single-photon microscope that enables recordings from genetically defined neurons at cellular resolution during fMRI. Using this approach, we find that neurons located close to blood vessels often show a negative relationship with the local BOLD signal, whereas neurons farther away display a more variable, often positive relationship. We further demonstrate that local neural activity can be linked to BOLD responses across distributed, connected brain regions. Together, these results provide insight into how vascular organization shapes fMRI signals and establish a powerful tool for bridging cellular and whole-brain measurements of brain function.

## Abstract (web summary)

Imaging of brain-wide vascular activity with BOLD fMRI in combination with calcium imaging at single-cell resolution using MRI-compatible components provides detailed insights into neurovascular coupling in mouse models.

## Main

Brain function emerges from the coordinated interactions of global brain areas and local neural circuits. Investigation of brain function therefore benefits from experimental methods that can probe neural activity throughout the whole brain, preferably with a high spatial and temporal resolution at the level of local neuronal circuits.

Widely adopted imaging methods, such as functional magnetic resonance imaging (fMRI), allow non-invasive, whole-brain recordings but compromise spatiotemporal resolution and source specificity for coverage and non-invasiveness. In particular, blood-oxygen-level-dependent (BOLD) fMRI measures neural activity indirectly through changes in blood oxygenation and volume driven by neurovascular coupling, a process with an inherent temporal delay^1,2^. The BOLD fMRI signal is therefore considered a slow, mean proxy of local brain activity, with a limited ability to resolve local neural-circuit dynamics^3^.

By contrast, opto-physiological recording methods, such as in vivo single- and multi-photon imaging, can capture the membrane voltages and Ca^2+^ levels of genetically defined neural circuits at cellular resolution^4,5^. Recent Ca^2+^ imaging studies in rodents have demonstrated the possibility of simultaneously monitoring thousands of individual cells at kHz sampling rates^6,7^. However, optical methods typically require invasive surgery to gain access to the brain^8,9^ and can simultaneously measure only up to a few cortical brain regions of interest^10^.

To bridge the gap between local neural circuit activity and the hemodynamic BOLD fMRI signal, researchers have attempted to combine optical imaging methods with fMRI. One common in vivo combined imaging approach (in vitro approaches have also been created^11^) has been to perform sequential fMRI and Ca^2+^ imaging in the same individual^12^. This approach allows for cross-session comparisons of averaged responses and facilitates the identification of potential spatiotemporal relationships between neural and vascular activity. However, because the data are collected separately, this approach cannot control for variability across trials, sessions or experimental environments^13,14^. This means that observed differences could be confounded by methodological variability, rather than reflecting biological effects, highlighting the need for a combined imaging approach that can capture neural and hemodynamic activity simultaneously.

In an effort to better understand the temporal relationship between neural circuit activity and BOLD fMRI, several research groups have developed methods for simultaneous optical and BOLD fMRI imaging^15–21^. For example, Schulz et al. used combined fiber photometry and BOLD fMRI to show that the BOLD fMRI signal not only reflects neural sources, but also relates to local slow oscillation Ca^2+^ waves in astrocytes^16^. More recently, Lake et al. expanded on combined fiber-photometry fMRI by developing a multi-fiber bundle to record simultaneous macro-scale Ca^2+^ and BOLD activity across the whole cortex^19^. Their results show that the BOLD fMRI signal across the cortex can be partially reconstructed from local neural-activity sources with high spatial precision, laying the foundation for a better understanding of the neural underpinnings of the BOLD fMRI signal.

These previous studies have demonstrated that combined optical and BOLD fMRI approaches offer a powerful way to explore neurovascular dynamics. However, fiber-based optical methods do not provide the spatial resolution needed to distinguish single neurons and instead group together (non-)neuronal cellular and neuropil (synaptic and dendritic) signals. To overcome this limitation, we developed an MRI-compatible single-photon microscope that can measure cellular-resolution Ca^2+^ activity of genetically defined neurons, without inducing artifacts, during whole-brain BOLD fMRI acquisition in awake mice. By providing cellular-resolution readouts of neuronal activity in the context of whole-brain functional imaging, our approach enables the direct comparison between the activity of genetically defined local neuronal populations and vascular responses across the rodent brain.

## Results

### Combined MRI and fluorescence microscopy enable simultaneous whole-brain fMRI and single-cell-resolution population imaging

**Fig. 1:**
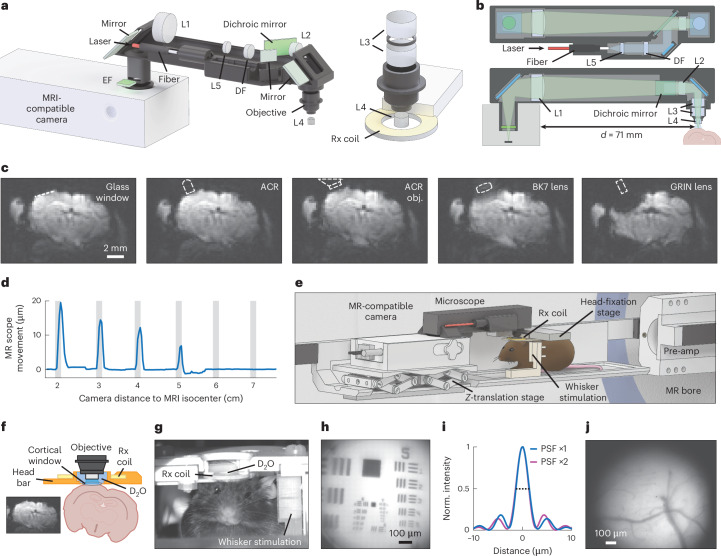
MRI-compatible microscope design for combined BOLD fMRI and calcium imaging. **a**, Render of the 3D-printed MRI-compatible microscope (left) and objective (right). Excitation light from an off-site laser is collimated with a double-convex lens (L5) and passed through a diffuser (DF). Fluorescent emission is collected by an objective consisting of three small diameter acrylic aspheric lenses (L3, L4). The collected light is relayed through two achromatic lenses (L1, L2), and projected onto the MRI-compatible CCD camera through an emission filter (EF). Rx coil, receive coil. **b**, Schematic of the excitation (blue) and emission (green) optical pathways. The distance between the CCD camera housing and the sample is ~71 mm. **c**, BOLD EPI of a mouse brain (in situ) with and without optical components placed close to the Ca^2+^ imaging site above the barrel cortex. The approximate locations of the optical components are indicated by dashed outlines. From left to right, glass window only, acrylic lens (ACR), acrylic lens and HDT180 objective, BK7 objective lens, gradient-index (GRIN) objective lens. **d**, Microscope displacement induced by RF excitation pulses with respect to the distance between the MRI-compatible camera and the isocenter of the B_0_ magnetic field. **e**, Complete MRI imaging setup including the microscope, a translational stage for positioning, the receiver coil, the whisker-stimulation apparatus, the head-fixation stage, the pre-amplifier (pre-amp) and the MRI scanner. **f**, Schematic of the implant, Rx coil, head bar and cortical window. Inset, BOLD EPI image with D_2_O immersion. **g**, Photo of the same implant setup showing a mouse fixed in the behavioral cradle. **h**,**i**, High-resolution image of a microscope calibration slide (**h**) and the simulated optical resolution of the microscope (**i**) with a full-width-half-maximum (dashed line) of 2.5 µm. **j**, Single fluorescence images of the barrel cortex taken with the MRI-compatible microscope.

Ca^2+^ imaging is typically performed with microscopes that consist of materials and electronics incompatible with the magnetic field of MRI scanners. To combine Ca^2+^ imaging and MRI, we designed a small MRI-compatible microscope that functions in the limited space of a standard small-animal MRI volume coil. The main components of our MRI-compatible imaging device include a CCD camera, fluorescence light excitation and imaging optics, and a custom three dimensional (3D)-printed microscope body (Fig. 1a,b).

To minimize artifacts and optimize the signal-to-noise ratio (SNR) of BOLD fMRI, we took several measures to ensure the homogeneity of the magnetic field and to avoid electromagnetic interference, without compromising fluorescent signal transmission. These include (**1**) shielding or positioning MRI-incompatible electronics outside the MRI’s magnetic field, (**2**) the use of MRI-compatible optical and body materials and (**3**) a custom microscope form factor to avoid camera displacement artifacts that occur during MRI gradient switching.

First, to ensure the signal quality of the BOLD fMRI images, we shielded any non-compatible electronics and materials and positioned these far from the magnetic field. For the excitation light, we coupled an external light source into a 400-μm optical fiber (Fig. 1a), which allowed us to place the fluorescence light source outside the magnetic field of the MRI scanner. In addition, we used a shielded MRI-compatible CCD camera to record the microscope’s fluorescent signal. This enabled us to place the camera close to the animal’s head inside the MRI bore, which is essential for high-resolution Ca^2+^ imaging.

Second, we tested all components of the microscope to avoid MRI artifacts. These included the optical lenses, microscope body and mouse head implant for in vivo imaging. We show the effect of different objective lenses, as well as the cortical window on the BOLD signal in an ex vivo mouse brain. We found that the magnetic susceptibility index of conventional borosilicate glass (BK7) and gradient-index lenses for volumes used in our microscope objective lead to substantial susceptibility signal loss owing to within-voxel through-plane variation of the B_0_ field (Fig. 1c; for a detailed review, see refs. ^12,22^). To resolve this issue, we designed the miniaturized microscope objective using readily available acrylic lenses (Fig. 1a,b). Although acrylic offered clear advantages for the objective, we decided not to use it for the cortical window, owing to concerns about durability and optical flatness. Instead, we used BK7 glass for the window and aligned it with the B_0_ field to minimize distortions^12^. The imaging window was positioned such that the surface normal was orthogonal to the B_0_ vector, thereby containing the susceptibility artifact to voxels immediately adjacent to the implant site. Our approach allowed us to record microscopic fluorescent images inside the MRI scanner with minimal compromise to the BOLD fMRI signal quality (Fig. 1c).

**Extended Data Fig. 1:**
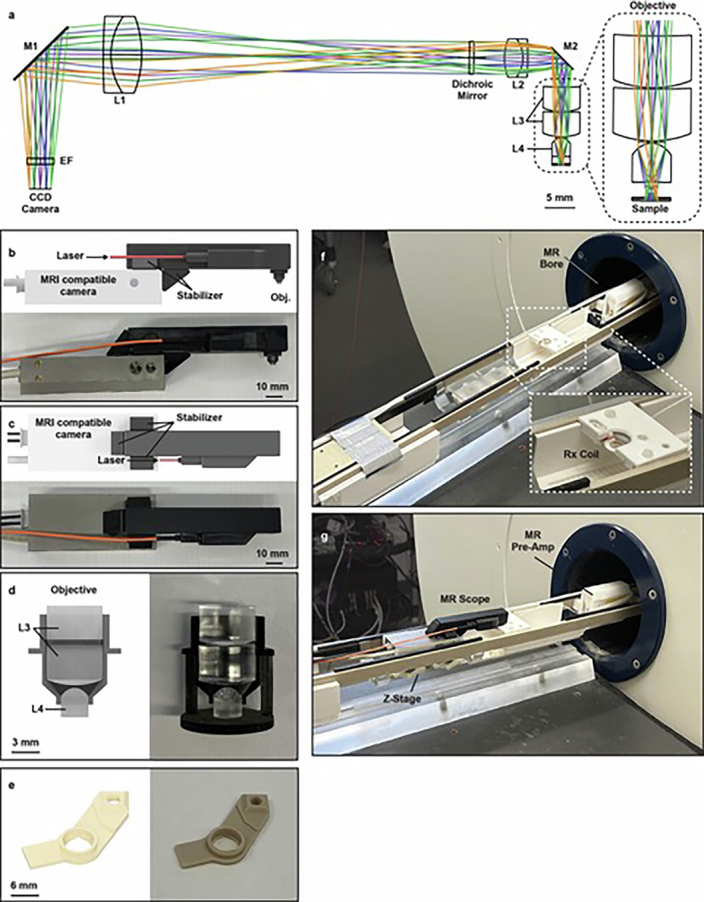
MRI-compatible microscope setup for combined BOLD fMRI and calcium imaging. **a**, Zemax simulation of the emission optical pathway. Fluorescent emission is collected by an objective consisting of three small diameter acrylic aspheric lenses (L3, L4). The collected light is relayed through two achromatic lenses (L1, L2), and projected onto the MRI-compatible CCD camera through an emission filter (EF). **b**, Side view of the MRI-compatible microscope. **c**, Top view of the MRI-compatible microscope. **d**, Cross-section of the objective consisting of three small diameter acrylic aspheric lenses (L3, L4). The real image (right) includes the support structure used for aligning the bottom lens (L4) during construction. **e**, The custom designed PEEK headbar. **f,g**, Picture of the complete imaging setup including the microscope, translational stage for focusing, and the MRI scanner. The inset in **f** shows the head fixation stage, and receiver (Rx) coil.

To further optimize the BOLD fMRI signal, we tested different materials for the microscope and objective body (Extended Data Fig. 1). We found that the 3D-printed, high-temperature-resistant epoxy adhesive featured sufficient printer resolution, durability, tensile strength and resistance to heat, resulting in more durable and stable microscope recordings. In addition, we found that SNR levels are comparable to other polymers containing black pigments (Extended Data Table 1).

Third, we observed micrometer-sized shifts in the microscope recordings during the radiofrequency (RF) MR excitation pulses, which we hypothesize could result from a combination of factors, including (**1)** Lorentz forces or eddy-current-induced vibrations owing to the slight magnetic susceptibility of the camera’s electronics and/or housing and (**2)** RF-induced coupling in internal circuits or cables. To avoid any possibility of microscope motion, we measured the minimum distance (*d*) needed between the edge of the camera and the center of the magnetic field (Fig. 1b). We found that *d* > 6 cm was sufficient to avoid any microscope displacement (Fig. 1d), leading us to elongate the microscope imaging pathway by adding a two-lens optical-light-path relay (Fig. 1b). This effectively increased the distance between the microscope CCD camera and the RF-excitation field to 7.1 cm, resulting in stable microscope recordings (Fig. 1b).

**Extended Data Fig. 2:**
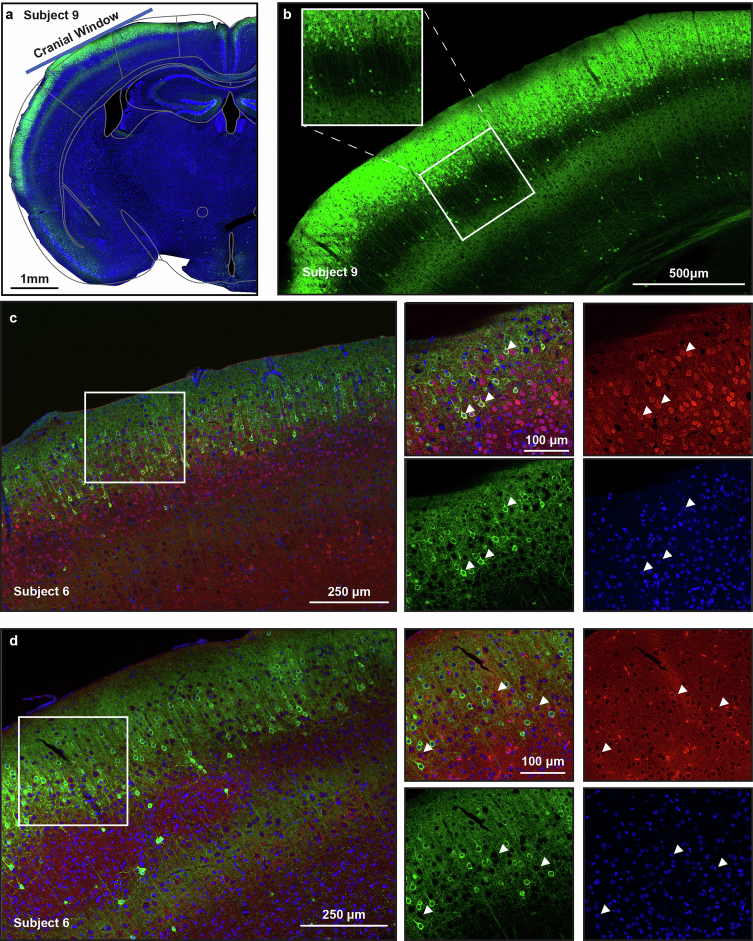
GCaMP6f-labeled cell-type verification. **a**, Coronal brain slice stained with DAPI (blue) and expression of GCaMP6f (green) in layer 2/3 of a Rasgrf-2A-Cre animal. The cranial window is shown as a blue line located above the SSp-bfd. **b**, Close-up of the same slice with a blow-up of the barrel fields. **c**, Immunohistological verification of different cell-types. Neurogranin positive cells are shown in red, GCaMP6f is shown in green, and DAPI in blue. **d**, Immunohistological verification of different cell-types. GAD65 + 67 positive cells are shown in red, GCaMP6f is shown in green, and DAPI in blue.

For in vivo Ca^2+^ imaging, we implanted transgenic mice (Rasgrf2-2A-dCre^23^ crossbred with Ai148D) expressing the Ca^2+^ indicator GCaMP6f in cortical layer 2/3 with a round cortical window (3 mm diameter) above the barrel cortex (Extended Data Fig. 2; SSp-bfd, anteroposterior coordinate (AP) = 2.0 mm, lateral coordinate (LR) = 3.0 mm).

To position and focus the MRI-compatible microscope inside the MRI bore, we custom designed a micrometer-resolution translation stage out of aluminum and 3D-printed resin (Fig. 1e). We then assembled the MRScope, translational stage, a custom 3D-printed whisker-stimulation apparatus and the housing into a cradle that fit inside the bore of a small-animal MRI scanner (Fig. 1e).

**Extended Data Fig. 3:**
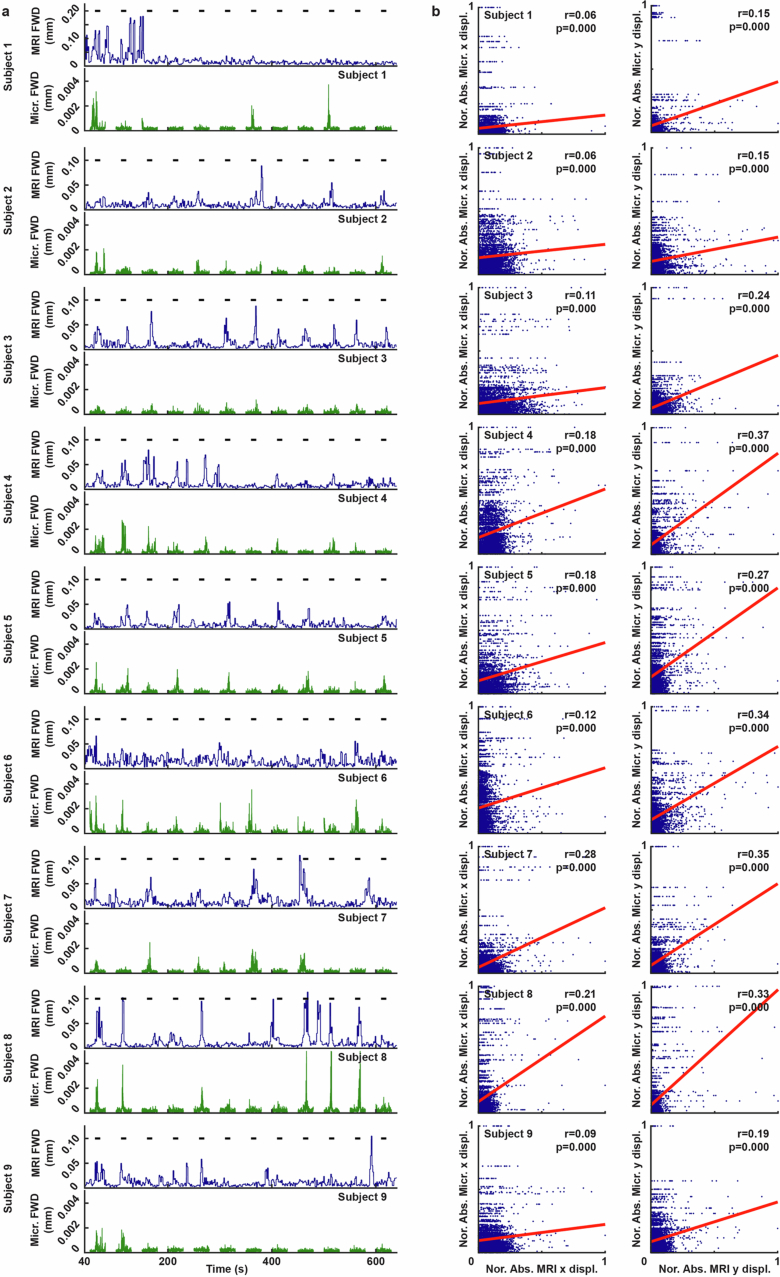
Motion parameters for individual animals. **a**, Framewise displacement (FWD) for MRI (top) and microscopy (bottom). Movement traces represent FWD calculated as the sum of absolute translational displacements in the x, y, and z directions, plus rotational displacements converted to millimeters using a fixed radius for MRI (top), and the sum of absolute x and y displacements for microscopy (bottom). Traces are plotted across time for individual sessions. Black lines indicate whisker stimulation duration. **b**, Scatter plots showing the correlation between the normalized MRI-based and microscopy-based motion estimates. Linear regression lines are overlaid in red. Correlation coefficients (Pearson’s *r*) and associated *p*-values are reported for each comparison.

**Extended Data Fig. 4:**
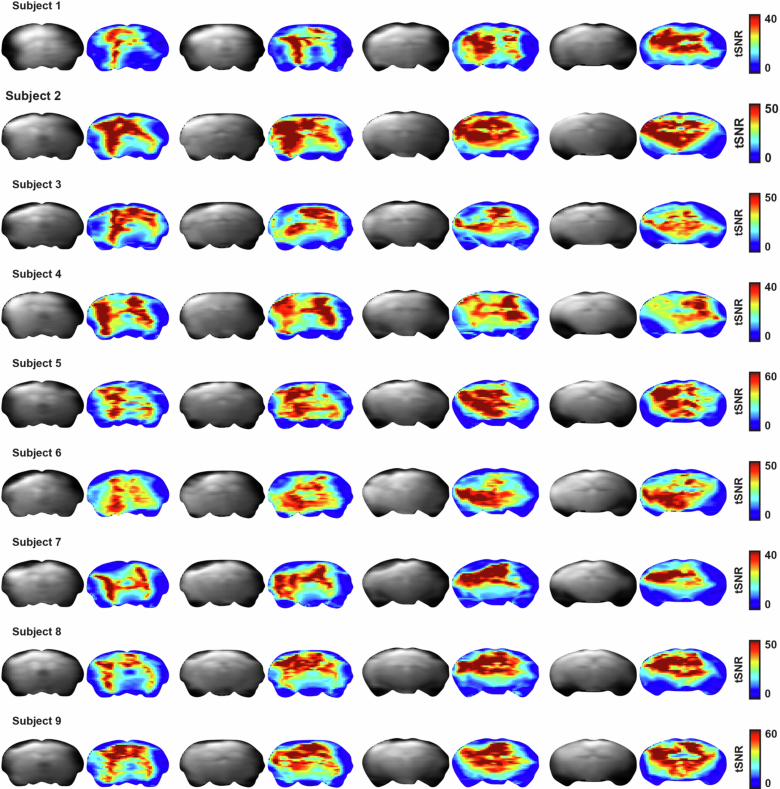
Temporal SNR for individual animals. Temporal signal-to-noise ratio (tSNR) maps of the BOLD fMRI data and corresponding mean BOLD images for anatomical reference. tSNR was calculated voxelwise as the mean signal over time divided by the standard deviation over time. Higher tSNR values indicate a more reliable temporal signal within each voxel.

To prepare animals for the awake imaging experiments, we started with a habituation period (>10 days) during which we handled all animals and slowly introduced them to the MRI environment and stimulation protocol. After animals became familiar with the experimental procedure and the MRI sound, we initiated the experimental phase. During imaging experiments, we individually head-fixed each mouse in the MRI cradle (Extended Data Figs. 3 and 4), mounted the MRI receiver coil above the cortical window, positioned and focused the MRScope and added D_2_O (D-content, 99.9%) as an immersion medium between the acrylic lens objective and the cortical window (Fig. 1f,g). Designing an acrylic water immersion objective was crucial because the susceptibility borders between air and tissue resulted in a large signal void owing to local field inhomogeneities and thus artifacts in the MR image.

The field of view (FOV) of the MRI-compatible microscope covered a large cortical area across the barrel cortex and allowed us to acquire fluorescence images with single-cell resolution in the awake mouse barrel cortex inside the MRI scanner (FOV, 1.45 mm^2^; pixel resolution, 1.1 μm; objective NA, 0.363; optical resolution, 2.5 μm ± 0.24 μm (full-width-half-maximum, mean ± s.d., *n* = 10); Fig. 1h–j), without any noticeable BOLD fMRI artifacts or decreases in SNR.

### Simultaneous recordings of BOLD fMRI and cellular-resolution calcium activity

**Fig. 2:**
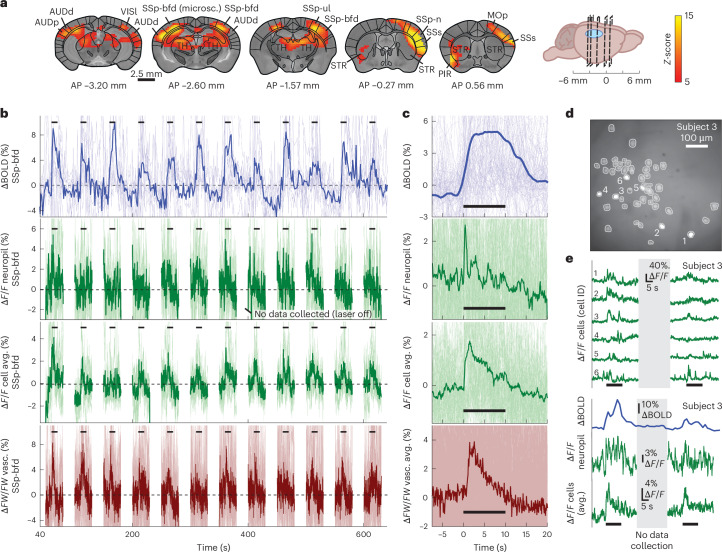
Simultaneous BOLD fMRI and calcium imaging of neural population activity at single-cell resolution. **a**, Statistical parametric map (fixed effects, two-tailed *t*-test, *z*-score ≥ 3.1, FWE *P* ≤ 0.00001, *n* = 9) generated from the ge-EPI BOLD fMRI images acquired during contralateral whisker stimulation. The schematic (right) shows the location of each frame relative to the cortical window. AUDd, dorsal auditory cortex; AUDp, primary auditory cortex; MOp, primary motor cortex; PIR, piriform cortex; SSp-n, somatosensory orofacial domain; SSp-ul, somatosensory upper limb domain; VISI, primary visual cortex. **b**, BOLD fMRI (top; *n* = 9), neuropil and cellular population Ca^2+^ activity (middle; *n* = 5), and vascular diameter (bottom; *n* = 9) response in the somatosensory barrel field (SSp-bfd), averaged over animals. Individual sessions are plotted in the background to show variability between subjects. Black lines indicate the duration of whisker stimulation. Interruptions in data sampling result from the laser-off period. F, fluorescence intensity; FW, full vessel width at half max. **c**, Evoked response to contralateral whisker stimulation, averaged over trials. Traces in the background show individual trials per subject. **d**, Cell map from one subject, extracted with CNMF-E^28^. Numbered cells correspond to the individual activity traces shown in **e**. **e**, Example activity traces for individual cells, BOLD fMRI, neuropil and the neural population for a single mouse during whisker stimulation. ∆*F*/*F* neuropil and ∆*F*/*F* cells indicate the ∆*F*/*F* measured in the SSp-bfd neuropil and extracted SSp-bfd cells, respectively. Black lines indicate whisker stimulation. The neural activity corresponds to the extracted cells highlighted in **d**. Interruptions in data sampling result from the laser-off period.

To test and validate our combined imaging approach, we simultaneously recorded population Ca^2+^ signals and BOLD fMRI responses in awake mice during whisker stimulation (Fig. 2). We presented each mouse with 12 trials (block design) consisting of 10 s pneumonic (air) stimulation at 3 Hz (40 ms duty cycle) to the full whisker pad located contralateral to the MRI-compatible microscope.

**Extended Data Fig. 5:**
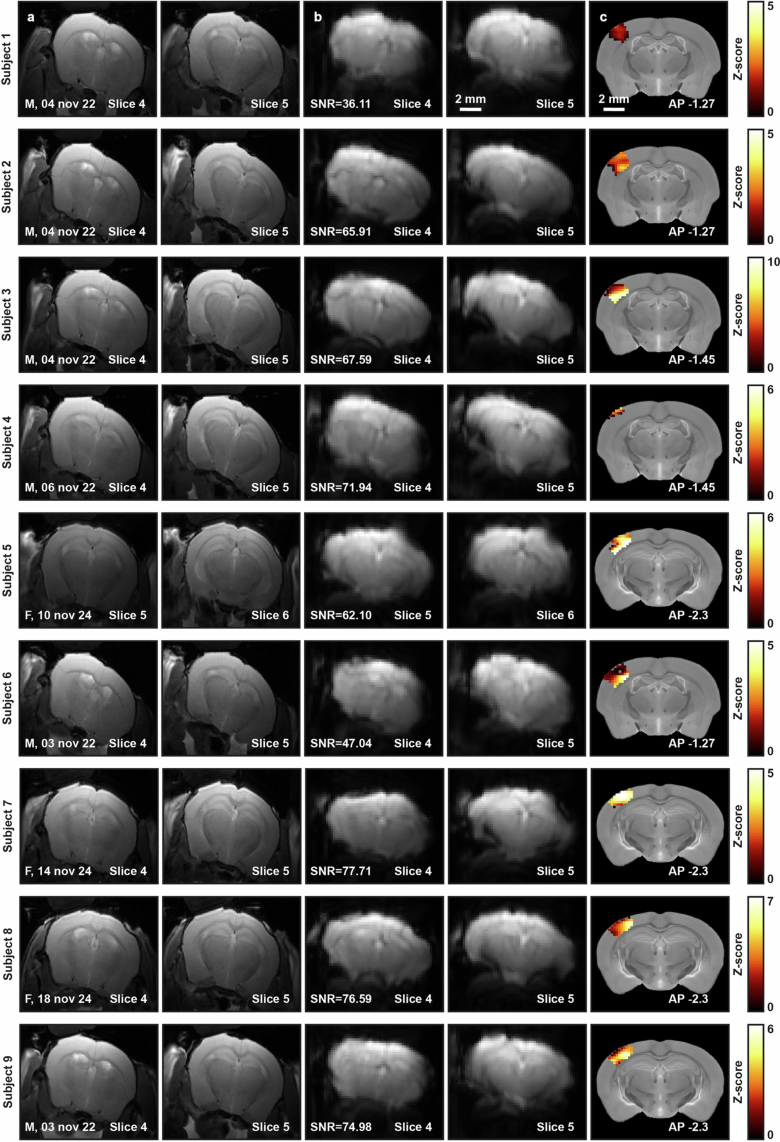
Coronal MRI slices around the glass-window implant used for optical imaging, and General Linear Model based statistical maps of the SSp-bfd for individual animals. **a**, Unprocessed anatomical images of the mouse brain with brain implant and MRI-compatible microscope installed. **b**, The single-shot gradient-echo (GE) echo planar imaging (EPI) for the same slices as depicted in **a**. The signal-to-noise ratio (SNR) was calculated as the ratio of the mean signal intensity within a manually defined signal region of interest (ROI) to the standard deviation of the signal within a separate background (noise) ROI, computed across all timepoints. **c**, The GLM statistical parametric map for each animal (z-score ≥ 3.1, FWE *P* ≤ 0.000001) shows significantly activated voxels in the SSp-bfd for each mouse.

To identify BOLD fMRI voxels that were sensitive to stimulation, we used a generalized linear model (Extended Data Fig. 5). We found robust BOLD activation across several cortical and subcortical regions in response to unilateral whisker stimulation (Fig. 2a; *z*-score ≥ 3.1, family-wise error-corrected (FWE) *P* ≤ 0.00001; *n* = 9, 6 males, 3 females). Consistent with previous work, the main regions exhibiting BOLD signal increase included regions commonly involved in somatosensation, such as the bilateral thalamus (TH), striatum (STR), secondary somatosensory area (SSs), orofacial domains and the whisker domains (SSp-bfd)^24–27^. The strong activation of auditory regions, including the bilateral primary and dorsal auditory cortex, could be explained by the audible pneumonic pressure generated during stimulation.

To demonstrate the sensitivity of our combined imaging approach, we extracted the activity time series for all mice from significantly activated SSp-bfd voxels located ipsilateral to the microscope recordings (Extended Data Fig. 5; *z*-score ≥ 3.1, FWE *P* ≤ 0.00001; *n* = 9, 6 males, 3 females). We found that the BOLD fMRI activity showed strong stimulus-evoked potentials in the ∆BOLD signal (Fig. 2b,c). The activity also revealed a high degree of temporal signal fluctuations, most likely owing to spontaneous neural activity and residual movement^12,14^. The trial-averaged response shows a well-defined hemodynamic response (~4.9% signal change; time-to-peak 80% (TTP_80%_): 1.7 s; Fig. 2c), which is in line with previous studies using a similar paradigm^12,25^.

Next, we extracted the ∆*F*/*F* Ca^2+^ activity for each individual cell (236 layer 2/3 SSp-bfd neurons; 46 ± 14 per animal (mean ± s.d.); *n* = 5, 4 males, 1 female; Fig. 2d,e), as well as the overall background signal (neuropil; *n* = 5, 4 males, 1 female; Fig. 2e) from our MRI-compatible microscope recordings using CNMF-E^28^. In addition, we averaged the time series of individual cells, resulting in one mean population activity signal per animal (Fig. 2e). To prevent possible vascular modulation and ensure that high-quality data were obtained during trials, we switched off the laser during rest periods to minimize potential thermal effects on the tissue^29^. For both the population and neuropil ∆*F*/*F* signal, we observed a strong stimulus-evoked signal on the subject level (Fig. 2b,c) and for the trial-averaged time series (neuropil: ~2.7% signal change; TTP_80%_: 0.2 s; population activity: ~1.7% signal change; TTP_80%_: 1.1 s; Fig. 2c).

Finally, we extracted the stimulus-evoked changes in vessel diameter from the microscope recordings (*n* = 9, 6 males, 3 females; Fig. 2b,c). The resulting time series showed robust stimulus-evoked vascular responses (~3.8% signal change; TTP_80%_: 1.4 s; Fig. 2c). Together, these results show that our combined approach enables simultaneous measurements of BOLD fMRI, vascular dilation and single-cell population activity without any noticeable artifacts.

### Neuronal responses exhibit diametric activation patterns depending on their distance to the local vasculature

**Fig. 3:**
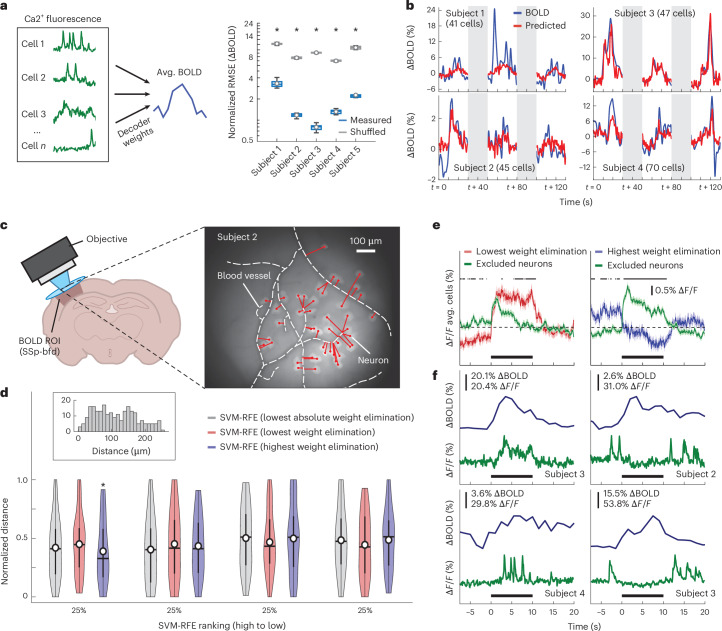
Spatial location of neurons with respect to the vasculature has an effect on the predictive relationship between BOLD and single-cell calcium activity. **a**, Left: SVM decoding was used to quantify how well local SSp-bfd neural activity predicted the BOLD fMRI signal. Right: the NRMSE between the measured and predicted ∆BOLD signal is shown. Boxplots show the NRMSE of the 10× cross-validated outcomes for each animal (*n* = 5). The center line represents the median, and the circle represents the mean. The minimum and maximum of the box extend to the 25th and 75th percentiles, whiskers extend to all data points. The significance test shows the difference in performance between measured and shuffled data (two-sided Wilcoxon signed rank test; *P ≤ 0.005). **b**, Example predictions of the decoding approach for the SSp-bfd ∆BOLD signal. Gray boxes indicate the laser-off period. **c**, Blood vessels were manually annotated, and neuron-vascular proximity was defined as the Euclidean distance between the neuron and the nearest point on the blood vessel. **d**, Neuron-vascular proximity was quantized on the basis of the SVM recursive feature elimination (SVM-RFE) decoding procedure using three ranking criteria. The center line represents the median, and the circle represents the mean. Whiskers extend from the 25th to the 75th percentiles, and the fine whisker extends to all data points. The width of the plot is proportional to the number of data points with the corresponding value. The significance test shows the difference between the distance values within each quantile (two-sided Wilcoxon rank-sum test; *P ≤ 0.05). No correction for multiple comparisons was applied. Inset, distance histogram. **e**,**f**, Trial average ∆*F*/*F* Ca^2+^ activity of the 25% highest ranked cells (red) versus the lowest ranked 75% cells (green; **e**), and example ∆BOLD and ∆*F*/*F* Ca^2+^ activity traces (**f**). Shaded areas represent the SEM across cells. Black lines indicate whisker stimulation. Significance test shows difference between traces (two-sided two-sample *t*-test; *P* ≤ 0.001).

Next, we explored the potential of our approach to investigate the local relationship between the single-cell Ca^2+^ activity and the BOLD fMRI responses. To quantify the predictive information of the SSp-bfd neural population with respect to the local BOLD fMRI signal, we used a decoding approach based on support vector machines (SVMs). For this analysis, we used the ∆*F*/*F* Ca^2+^ activity traces from individual neurons to predict the average ∆BOLD fMRI signal in the SSp-bfd (Fig. 3a).

Our results demonstrate that the normalized root mean squared error (NRMSE) of all predicted responses is lower than the ones of the temporally shuffled baseline model (Fig. 3a,b; two-sided Wilcoxon signed rank test; *P* ≤ 0.005). This shows that the neural population activity contains information about the local ∆BOLD fMRI signal, and confirms the validity of our decoding approach.

**Extended Data Fig. 6:**
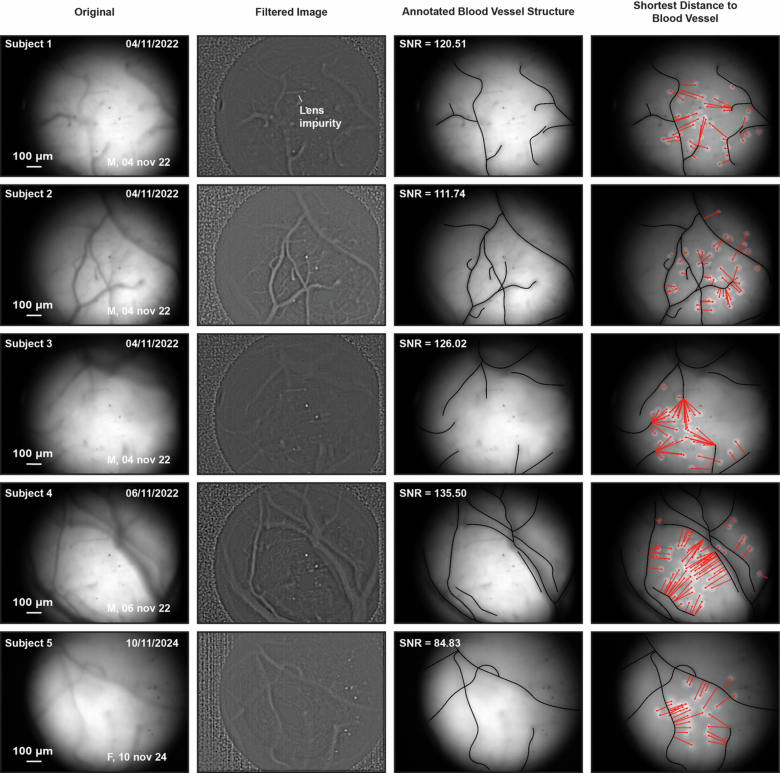
Annotation of the blood vessel patterns for individual animals. The neural-vascular proximity was estimated based on the manual annotation of the vessel structure in the region of interest for each mouse. We used the time-averaged microscopic field (first column) and the band-passed filtered (100-150 Hz) microscopic field (second column) to accurately distinguish the vessel structure (third column). The neural-vascular proximity was calculated as the minimal Euclidean distance between the center of the cell and the vascular structure (fourth column). The signal-to noise ratio (SNR) was calculated as the ratio of the mean signal intensity within a manually defined signal region of interest (ROI) to the standard deviation of the signal within a separate background (noise) ROI, computed across all timepoints.

However, population Ca^2+^ imaging provides not only information about the neural activity and vasculature structure, but also their spatial arrangement. To investigate the effect of neuron location in predicting the local BOLD fMRI signal, we compared the Euclidean distance between the center of each neuron and the annotated vascular structure (Fig. 3c and Extended Data Fig. 6) to the contribution of each neuron to predict the BOLD fMRI signal. To estimate the contribution of each neuron, we used SVM recursive feature elimination^30,31^ to rank each neuron on the basis of its predictive information, and compared three ranking criteria: lowest absolute decoder-weight elimination, lowest decoder-weight elimination and highest decoder-weight elimination.

**Extended Data Fig. 7:**
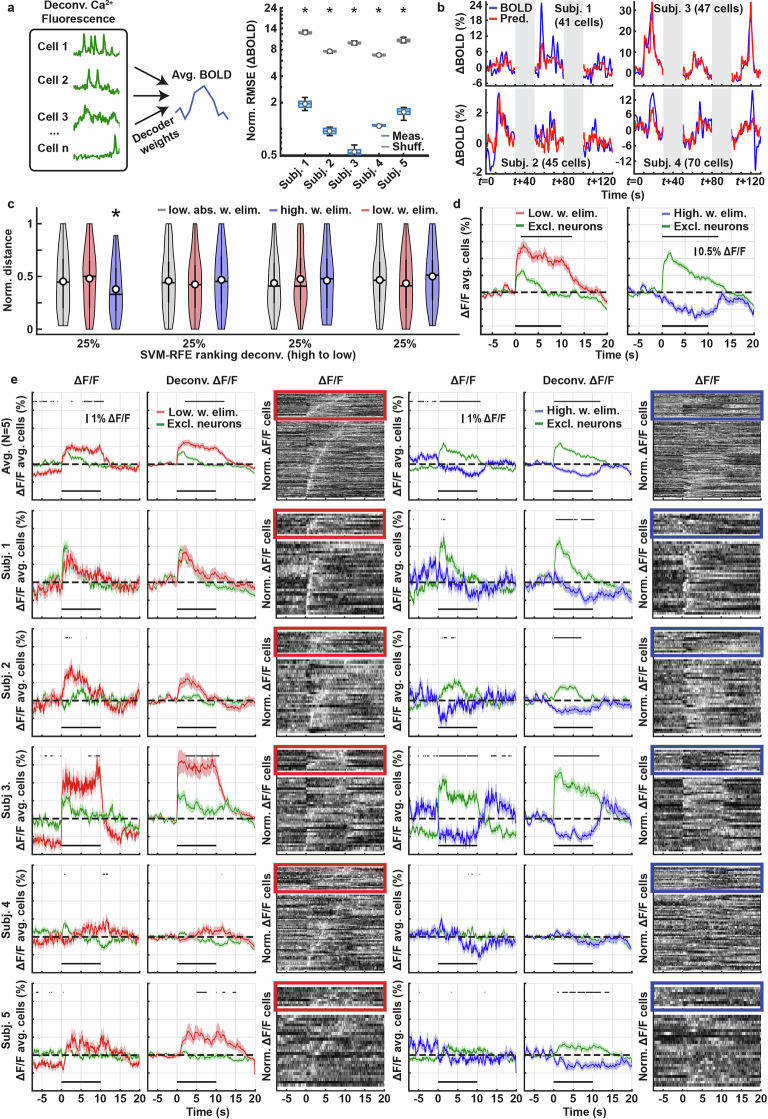
Neuron–vascular proximity influences the predictive relationship between BOLD fMRI and single-cell calcium activity independently of ∆F/F processing and remains stable across neurons. **a**, Schematic of the support vector machine (SVM) decoding approach used to quantify predictive information of local neural population activity in SSp-bfd for the BOLD fMRI signal (left). In contrast to Fig. 3, deconvolved ∆F/F Ca^2+^ activity extracted using the CNMF-e pipeline (constrained FOOPSI, decay_time = 0.4; (P. Zhou et al. 2018)) was used. Right, normalized root mean square error (NRMSE) between measured and predicted ∆BOLD fMRI signal. Boxplots show tenfold cross-validated NRMSE per animal (N = 5, 4 male, 1 female). Center line, median; circle, mean; box, 25th–75th percentiles; whiskers, full range. Performance exceeded shuffled data (two-sided Wilcoxon signed rank test; P ≤ 0.005). **b**, Example ∆BOLD fMRI predictions for each subject. Gray boxes indicate laser-off periods. **c**, Neuron–vascular distance quantiles based on SVM recursive feature elimination (SVM-RFE), using three ranking criteria. Deconvolved ∆F/F Ca^2+^ activity was used. Overlap between highly ranked neurons and raw ∆F/F rankings was ~74%. Center line, median; circle, mean; box, 25th–75th percentiles; whiskers, full range; width reflects data density. Distance differed across quantiles (two-sided Wilcoxon rank-sum test; * = P ≤ 0.05). No correction for multiple comparisons was applied. **d**, Trial-averaged ∆F/F Ca^2+^ activity of top 25% ranked cells (red) versus remaining cells (green). Shaded areas indicate SEM across cells. Black lines indicate whisker stimulation. Traces differed significantly (two-sided two-sample t-test; P ≤ 0.001). **e**, Same analysis for individual mice, showing raw ∆F/F (left) and deconvolved ∆F/F (middle). Traces differed significantly (two-sided two-sample t-test; P ≤ 0.001). Right, raster plot of all neurons ranked by SVM-RFE contribution. Frame highlights the top 25% most informative neurons for lowest weights elimination (red) or highest weights elimination (blue).

We found no difference in spatial location compared with the whole population when neurons were ranked using the absolute predictive strength (lowest absolute decoder-weight elimination; Fig. 3d). However, including the sign of the decoder weights in the ranking criteria revealed a difference in activity patterns between cells, which was dependent on their location with respect to the vasculature (Fig. 3d–f and Extended Data Fig. 7). We found that neurons with a strong response to contralateral whisker stimulation were exclusively assigned positive decoder weights (lowest decoder-weight elimination; Fig. 3e,f), and showed a heterogeneous pattern in their distance from the vasculature that did not differ from the overall distribution (Fig. 3d; two-sided Wilcoxon rank-sum test; *P* = 0.8919). By contrast, neurons that were assigned negative decoder weights (highest decoder-weight elimination) showed a decreased response during whisker stimulation (Fig. 3e,f), and were located significantly closer to the vascular structure (Fig. 3d; two-sided Wilcoxon rank-sum test; *P* = 0.0440).

These results show that subpopulations of neurons in layer 2/3 of the SSp-bfd contain diametric information about the vascular BOLD fMRI response. In addition, we leveraged the spatial component of the MRI-compatible microscope recordings to show that the direction of the neuronal response was dependent on the distance to the local blood vessel structures. In the following section we will present a second experimental approach of how our technique enables researchers to investigate the properties of local circuit computation.

### From local population measurements to large-scale recordings of brain connectomes

Local neuronal activity (for example in the SSp-bfd) is influenced by inputs from multiple brain regions through direct and indirect afferent connections^32,33^. To fully understand local computation, it is therefore critical to monitor activity in connected areas that are often distributed throughout the brain. In this section, we demonstrate how our combined Ca^2+^ fMRI BOLD imaging approach can be used to examine the predictive information of the hemodynamic BOLD fMRI activity from different regions with respect to local population Ca^2+^ activity in the SSp-bfd.

**Fig. 4:**
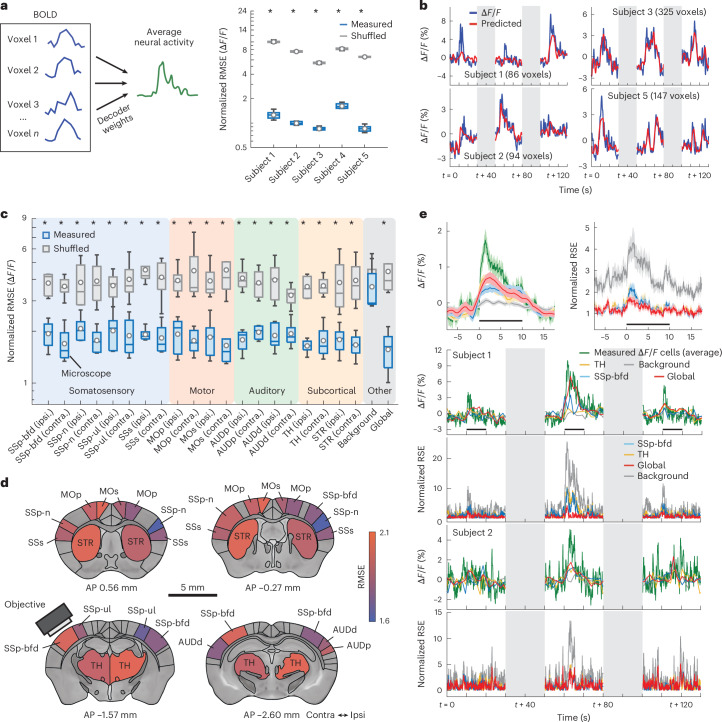
Predictive power of BOLD on the SSp-bfd population activity varies across brain regions. **a**, Left: SVM decoding was used to predict the SSp-bfd BOLD signal from local Ca^2+^ population activity. Right: The NRMSE between measured and predicted ∆*F*/*F* population activity is shown. Boxplots show NRMSE from 10× cross-validation for each animal (*n* = 5). Center line, median; circle, mean; box, 25th–75th percentiles; whiskers, full range. Performance differed between measured and shuffled data (two-sided Wilcoxon signed-rank test; *P ≤ 0.005). **b**, Example predictions of the SSp-bfd BOLD signal from the Ca^2+^ population activity. Gray boxes indicate laser-off period. **c**, NRMSE between the measured and predicted SSp-bfd ∆*F*/*F* population activity for several areas. Boxplots show the NRMSE of the 10× cross-validated outcomes for each animal (*n* = 5). The center line represents the median, and the circle represents the mean. The minimum and maximum of the box extend to the 25th and 75th percentiles, and whiskers extend to all data points. Significance test shows the difference in performance between measured and shuffled data (two-sided Wilcoxon rank-sum test; *P ≤ 0.01). MOs, secondary motor cortex; SSp-n, somatosensory orofacial domain; ipsi., ipsilateral; contra., contralateral. Laterality indicated with respect to stimulation. **d**, Magnitude map illustrating the predictive relationship between ∆BOLD and neural population activity. **e**, Example predictions and corresponding NRMSE of the SSp-bfd ∆*F*/*F* population activity for several brain areas and background. The trial average is shown at the top, and shaded areas represent the SEM. Gray boxes indicate the laser-off period. Black lines indicate whisker stimulation.

As before, we utilized a decoding approach based on SVM. However, in contrast with the previous analysis, we used the significantly activated voxels in the SSp-bfd (*z*-score ≥ 3.1, FWE *P* ≤ 0.00001) to predict the average ∆*F*/*F* neural population activity. Our results show that the NRMSE of all predicted responses fall far below the temporally shuffled baseline model (Fig. 4a,b; two-sided Wilcoxon signed-rank test; *P* ≤ 0.005), and demonstrates that the ∆BOLD fMRI signal contains information about the local neural population activity.

Next, we extended the approach to different brain regions that have previously been implicated in whisker stimulation (Fig. 2a)^32,34^. To avoid overestimating the amount of information for larger anatomical regions owing to overfitting, we applied PCA to decrease the number of input dimensions (20 components explained ≥ 75% of the variance for all areas). As control conditions, we also assessed classification performance on the basis of global and background activity. For global activity, we selected all voxels that contained brain activity and again applied PCA to decrease the number of input dimensions (20 components explained ≥75% of the variance). To estimate the background activity, we randomly selected 50 voxels located outside the brain mask, where no neurovascular signal is expected, and applied PCA (20 components explained ≥99% of the variance).

**Extended Data Fig. 8:**
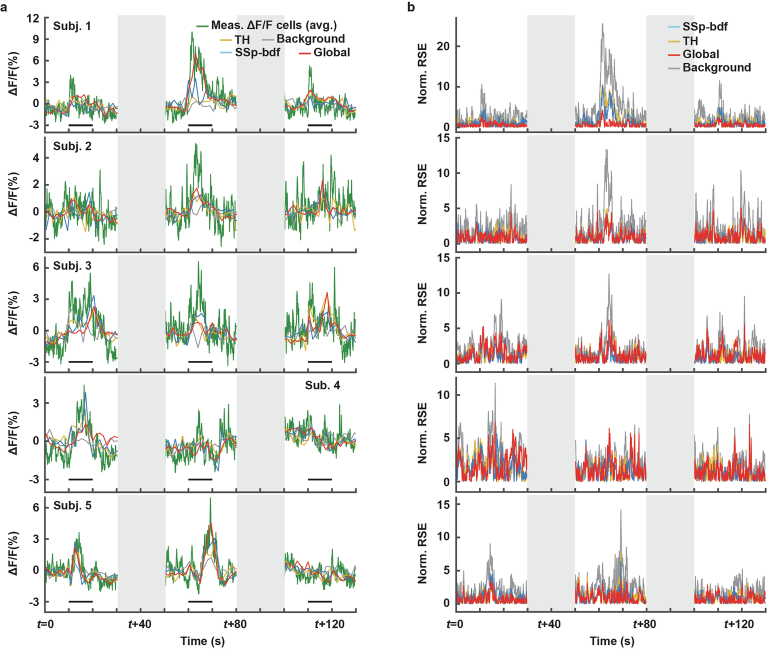
BOLD fMRI prediction of the SSp-bfd ∆F/F population activity. **a**,**b**, Example predictions of the SSp-bfd ∆F/F population activity (**a**) and corresponding normalized root square error (**b**; NRSE) for different brain areas and background noise. Results are plotted for individual mice. Gray boxes indicate laser-off period. SSp-bfd = somatosensory barrel fields, TH = thalamus.

**Extended Data Fig. 9:**
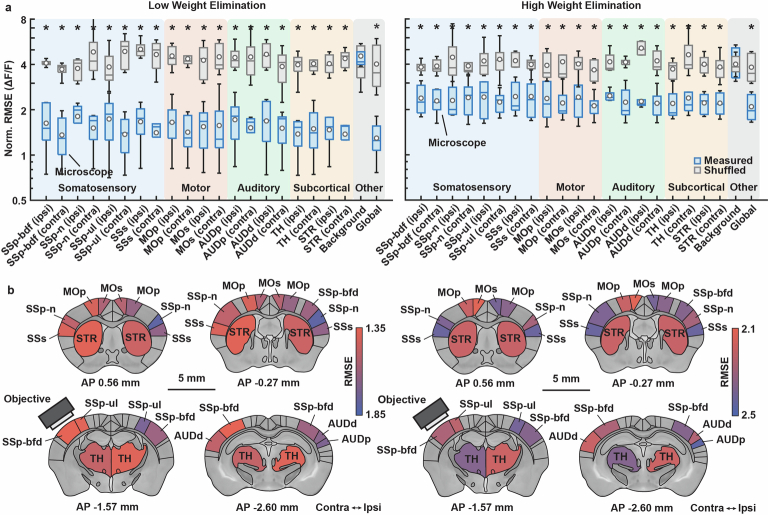
Predictive power of BOLD fMRI on the SSp-bfd population activity varies across different brain regions involved in whisker stimulation for subpopulations determined by SVM-RFE. **a**, NMRSE between the ∆F/F and predicted ∆F/F population activity for areas involved in whisker stimulation. Subgroups are determined by the analysis described in Fig. 3, and include both the low (left) and high (right) weight elimination subgroups. Boxplots show the NRMSE of the ten times cross-validated outcomes for each animal (N = 5). The central line represents the median, the circle represents the mean. The minimum and maximum of the box extend to the 25th and 75th percentiles, whiskers extend to all data points. We tested the difference in performance between measured and shuffled data using a Wilcoxon rank-sum test (Wilcoxon rank-sum test; * = *P* ≤ 0.05). AUDd = dorsal auditory cortex, AUDp = primary auditory cortex, MOp = primary motor cortex, MOs = secondary motor cortex, SSp-bfd = somatosensory barrel fields, SSp-n = somatosensory orofacial domain, SSp-ul = somatosensory upper limb domain, SSs = secondary somatosensory area, STR = striatum, TH = thalamus. Contralateral and ipsilateral indicated with respect to the whisker stimulation. **b**, Coronal magnitude map of the mouse brain illustrating the predictive relationship between the ∆BOLD and neural population activity based on the NRMSE described in **a**.

Our results show that the local neural population activity in the SSp-bfd can be predicted better from the ∆BOLD fMRI signal across the cortical and subcortical regions involved in whisker stimulation^32,34^ as compared with the temporally shuffled baseline model (Fig. 4c–e; two-sided Wilcoxon rank-sum test; *P* ≤ 0.01; Extended Data Fig. 8). When we visualized the performance of each region through a magnitude map (Fig. 4d and Extended Data Fig. 9), we can observe the spatial layout of areas and their predictive power. We did not observe significant differences between regions contralateral and ipsilateral to the stimulation (two-sided Wilcoxon rank-sum test; *P* = 0.18).

Together, these results demonstrate the possibilities of simultaneous global BOLD fMRI and cellular-resolution Ca^2+^ imaging. We show that different cortical and subcortical regions contain relevant predictive information about neural activity in the SSp-bfd. These regions are consistent with previous accounts of the mouse whisker system^24–27^ and the established anatomical connections between these regions^32,34^.

## Discussion

In this work, we developed an MRI-compatible microscope that enables the simultaneous imaging of single-cell Ca^2+^ activity during brain-wide BOLD fMRI acquisition. In previous work, several researchers have combined optical imaging methods, such as fiber-photometry-based Ca^2+^ imaging, with BOLD fMRI^15–17,19–21^. However, owing to the limited spatial resolution of fiber-based methods, (non-)neuronal cellular and neuropil signals are grouped together. In contrast to optical methods, combined electrophysiology and BOLD fMRI allows neural signals to be measured with single-cell resolution during BOLD fMRI acquisition, and several groups have used this method to investigate the neural underpinnings of the BOLD fMRI signal^35^. However, compensating for the electromagnetic interference inside the MRI scanner is costly and technically demanding. In addition, several key questions about the specific interactions between cell populations, such as excitatory and inhibitory neurons or glial cells and the BOLD fMRI signal, are difficult to address without the genetic targeting that Ca^2+^ imaging methods offer^36–39^. Our approach addresses these limitations by enabling genetic targeting while remaining cost-efficient and easy to implement, with a microscope that is simple to build and install in the MRI scanner.

We observed that highly predictive neurons closer to the vasculature exhibited a negative neural response during whisker stimulation, contrasting with the positive vascular BOLD fMRI activity. A possible explanation for the negative activity of cells located near blood vessels is their connectivity with specific subtypes of vasoactive interneurons. Previous studies have found that inhibitory neurons are involved in neurovascular coupling^40–44^, through the release of neurotransmitters that exert strong vasoactive effects when released in close proximity to the vasculature (vasodilatory VIP^+^ (ref.^45^); vasoconstricting SOM^+^ (ref. ^45,46^). This suggests that excitatory neurons exhibiting a negative relationship with local BOLD fMRI activity could be embedded in circuits that integrate vasoactive interneurons. Our approach enables future studies to investigate the relationship between the hemodynamic response and specific, genetically targeted, inhibitory subclasses at the single-cell level. Moreover, the microscope can be readily adapted for future experiments to support multicolor imaging of two genetically distinct cell populations^47,48^, or to integrate optogenetic stimulation during calcium imaging^49,50^.

Our results also show that the neural population activity in the SSp-bfd can be predicted with a high accuracy from the BOLD fMRI activity in different regions of the mouse brain. This finding is consistent with previous studies in that regions implicated in whisker stimulation^24–27^, as well as anatomically connected areas^32,34^, contained information about the local neural activity in the SSp-bfd. Using these methods, future studies can now investigate whether regions across the brain contain distinct predictive information about diverse local neuronal subsets that differ in their genetics or projection specificity. In addition, the suitability of our microscope to enable fMRI experiments in awake rodents means that these studies can be conducted during more natural behavioral paradigms.

Over the past years, a wide range of (imaging) techniques have been developed to investigate neurovascular function, each offering distinct advantages and limitations. Optical imaging approaches, such as wide-field calcium and intrinsic signal imaging, enable high-resolution recordings of cortical activity and hemodynamics^51^. These optical methods offer high spatial and temporal resolution and have been instrumental in the precise mapping of local neural and vascular responses during sensory stimulation^52,53^ and cognitive tasks^54–56^. However, their restricted field of view and confinement to the cortical surface limit access to neural dynamics across distributed brain networks.

To overcome these limitations, early studies integrated optical imaging spectroscopy with fMRI to demonstrate the feasibility of acquiring fast optical signals alongside whole-brain BOLD measurements^57,58^. These studies paved the way for subsequent studies combining fMRI and other optical methods, such as calcium imaging, to investigate the spatiotemporal relationship between neural activity and the hemodynamic response^15–21^. By enabling the direct comparison between the neural and vascular response across the brain, these studies have improved our understanding of neurovascular coupling and have informed the interpretation of the BOLD fMRI signals in both animal models and people. Finally, recent studies have extended these methods to awake animals, enabling more naturalistic investigations of neurovascular brain function^12,59–61^. Compared with previous approaches, the methods described here offer a unique combination of spatial coverage and cellular resolution in awake animals. Although all-optical methods provide high-resolution views of local activity and vascular responses, they are typically constrained to superficial cortical areas and cannot easily capture distributed network dynamics. Conversely, fMRI enables whole-brain imaging but lacks access to the underlying neuronal sources that drive the hemodynamic signal. By integrating single-photon calcium imaging with fMRI in the same awake animal, our approach bridges this gap and allows for the direct measurement of neuronal population activity while capturing the brain-wide hemodynamic context. This provides a powerful platform to investigate how local neural activity relates to large-scale vascular and functional networks under more naturalistic, awake conditions.

Although the current approach represents a substantial advance in the simultaneous measurement of local neural activity and brain-wide hemodynamic signals in awake animals, several methodological improvements remain possible. For example, whereas MRI voxels were aggregated within anatomical regions such as SSp-bfd in the present study, future work could improve spatial interpretation by co-registering the microscope FOV with the MRI volume. This could be achieved using geometric alignment strategies that estimate the microscope’s position and orientation (pitch, yaw and roll) relative to the underlying brain anatomy. Furthermore, in this study we estimated vessel locations from the visible outlines of large pial vessels. This approach can be improved by co-registering these images with higher-resolution two-photon vascular maps acquired offline (that is, in a secondary experiment). Ongoing developments are also advancing the integration of two-photon imaging with fMRI, offering the potential for deeper, layer-specific insights into neurovascular structure and function^62^. Finally, in the current study we used a simple stimulation paradigm to probe the neurovascular activity. Future work could employ more complex designs (that is, graded or spatially varied stimuli) to systematically assess how neural activity shapes the BOLD response^25,63^. Combining this with behavioral tracking (pupil dilation, detailed motion metrics) would help capture brain-wide dynamics under more naturalistic conditions^61^.

In conclusion, a deeper understanding of the interactions between neurons and the vasculature is essential for more accurate interpretation of BOLD fMRI data in both animal and human studies^64–66^, particularly when combined imaging experiments are performed in awake animals^25,59,67,68^. Integrating the high spatiotemporal resolution of calcium imaging with the brain-wide coverage of BOLD fMRI will provide deeper insight into the mechanisms underlying local neurovascular dynamics and the principles that govern brain function across spatial scales.

## Methods

All procedures were authorized by the veterinary office of the Canton Zurich, Switzerland, and are in agreement with the guidelines published in the European Communities Council Directive of 24 November 1986 (86/609/EEC).

### Animals

Mice were kept on a 12-hour/12-hour light–dark cycle with unlimited access to food and water. In total, 12 mice (8 male; 4 female) aged 4–6 months were used for experiments (25–35 g). Two animals were excluded because we observed no neuronal activity or low MRI SNR. One animal was excluded owing to substantial signal loss around the cortical implant. We collected all data from Rasgrf2-2A-dCre mice^23^ (Jackson Laboratory, stock no. 022864) cross-bred with Ai148D mice (Jackson Laboratory, stock no. 030328). The resulting mice expressed GCaMP6f in cortical layer 2/3 pyramidal neurons (Extended Data Fig. 2).

### Surgical procedures

#### Anesthesia

For all procedures, we administered buprenorphine (Bupaq, Streuli; 0.1 mg kg^−1^) and carprofen (Rimadyl, Zoetis; 4 mg kg^−1^) to all animals 30 min before anesthesia. We induced anesthesia with 5% isoflurane and maintained it at 1.5–2% throughout surgery. After the mouse was head-fixed in the stereotaxic frame (Kopf Instruments), hair was removed around the planned incision using depilation cream. During the whole surgical procedure, mice received 95% medical grade O_2_ (Pangas, Conoxia) through a face mask, while body temperature was kept at 37° Celsius using a temperature controller and a heating pad.

#### Cortical implant

Before opening the cranium, we injected 2% lidocaine between the skin and the skull as local anesthetic (Lidocaine, Streuli Pharma). After removal of the skin, we cleared the skull surface of soft tissue and debris. To increase the stability and attachment of the implant, we then applied a thin layer of phosphoric acid gel (etching gel; DMG Dental-Material) and removed it after 30 s with phosphate buffered saline (PBS, Sigma–Aldrich). In addition, we removed excess blood and applied a thin layer of Scotchbond universal adhesive (Scotchbond, 3M) to stabilize the exposed skull during the craniotomy and to create a barrier between the skull and the skin.

Finally, to gain optical access to the cortex, we performed a 3-mm-diameter craniotomy above the region of interest (SSp-bf; AP = 2.0 mm, LR = 3.0 mm), and sealed it with a single borosilicate glass cortical window (no. 1 thickness, 3 mm diameter; Fig. 1f). After fixing the cortical window in place using ultraviolet- and moisture-curable adhesive (Loctite 4305, Henkel), we placed a custom head-bar (polyether ether ketone, PEEK; Fig. 1f) around the window and attached it with Scotchbond.

#### Analgesic regime

For 3 days after each surgical procedure, animals received a subcutaneous (s.c.) injection of buprenorphine (Bupaq; Streuli, 0.1 mg kg^−1^) every 6 h during the light day cycle and in the drinking water (Bupaq; Streuli, 0.01 mg ml^−1^) during the dark day cycle, as well as an s.c. injection of carprofen (Rimadyl; Zoetis, 4 mg kg^−1^) every 12 h.

### Habituation procedure for awake animal combined imaging

Owing to the slow acquisition speed of BOLD fMRI, movement can cause signal displacement within an acquisition cycle. In addition, neuromodulatory factors such as stress have been shown to have a strong effect on neurovascular coupling, leading to confounding effects in the BOLD signal^69,70^. To minimize possible confounding factors, we implemented a rigorous habituation protocol (confounding motion factors are visualized in Extended Data Fig. 3).

After the surgery, we started to handle animals twice a day for a period of at least 3 min. Once animals were fully recovered and calm during handling, we initiated the habituation period. The duration of the habituation period depended on the behavioral performance of each individual animal and lasted a minimum of 10 days. During the first week, we restrained the animals in a mock MRI machine once a day for two to three days, followed by one day of rest. The duration of the head fixation started at 10 min, and increased stepwise by 5 min until the average duration of a full experimental session was reached (±30 min). We also introduced MRI scanner sounds during the fourth day of habituation, and gradually increased the sound pressure over four sessions until it reached the same level as during an MRI session (~95 dB). In addition, we used a blue ambient light to mimic the on–off cycles of the MRI-compatible microscope excitation light. After each habituation session, the animals received a small amount of reward, offered ad libitum for the duration of the handling.

Once the animal showed no discernible response to the head fixation, sound pressure level and excitation light^71^, we continued the habituation inside the MRI machine. The habituation sessions consisted of a functional scan that included the full stimulation protocol, as well as a structural scan. We used the framewise displacement (FWD)^72^ to evaluate habituation, and considered a session successful when the average FWD for a full session was below 0.05 mm and at least 50% of trials showed no excessive motion (FWD spikes > 0.075 mm). We chose the values for the FWD thresholds on the basis of the BOLD fMRI acquisition sequence, in which FWD spikes should not exceed more than 50% of the voxel size owing to spill over of the signal to nearby voxels. Once habituation was considered successful (>3 continuous days of sessions with movement below threshold), we initiated the combined imaging experiments (Supplementary Video 1).

### Optical components and assembly of the MRI-compatible microscope

We assembled all optics using custom 3D-printed housing (Loctite 3D 3860 HDT180, Henkel; Objet 30 Pro, Stratasys; Fig. 1a) and fixed them in place using low-staining optical glue (NOA61, Norland). We used durable and high-temperature-resistant resin to avoid warping owing to pressure and the surface heating of the camera. We found HDT180 to be the optimal trade-off between printer resolution, durability, tensile strength and heat-resistance (Extended Data Table 1 and Extended Data Fig. 1). However, repeated strain and heating of the plastic did result in moderate warping over a period of >2 years, leading to a decrease in SNR (Extended Data Fig. 6). To improve durability, the microscope body should be produced using stronger materials such as PEEK (polyether ether ketone; github.com/rlemubaghs/open_mrscope.git).

In the excitation pathway, blue light (wavelength *λ* = 488 nm) originating from the tip of a multimode fiber (FP400ERT, Thorlabs) coupled to an off-site laser (OBIS LX/LS Series, Coherent; Fig. 1b) is collimated using a 5-mm double convex lens (63-594-ink, Edmund Optics), and passes through a diffuser with a scatter angle of 10 degrees (edc-10, Rochester). The collimated light is combined with the optical path through a dichroic mirror (ET Dualband beamsplitter FITC/CY3, AHF) before it is focused on the brain surface through the objective.

In the imaging pathway, the emitted fluorescence is collected by the custom objective consisting of three small diameter acrylic aspheric lenses (15-271, Edmund Optics; 36-629, Edmund Optics). To minimize the vertical footprint of the microscope, the optical path is diverted 90 degrees by a mirror (350–700 nm imaging, AHF). Along the horizontal axis, light is relayed by two achromatic lenses (47-690-INK, Edmund Optics; AC080.020, Thorlabs). Finally, a second mirror reflects the image downwards through an emission filter (ET Dualband Emitter FITC/CY3, AHF) onto the MRI-compatible camera (MRC HighResolution, MRC Systems; 1,280 × 960 pixels at 5.3-μm pitch).

### Imaging parameters

#### Functional and structural MRI parameters

We acquired MRI images on a 7-T, 11-cm horizontal bore scanner (BioSpec 70/20 USR, BRUKER BioSpin MRI) with a BGA 9SHP (90 mm, max 750 mT/m gradient strength, slew rate 6,840 T m^−1^ s^−1^, BRUKER BioSpin MRI). The scanner was fitted with a RF RES 300 1H 089/072 QSN TR AD volume coil combined with an RF SUC 300 1H LNA AV3 surface coil to receive the radiofrequency signal (BRUKER BioSpin MRI). BOLD fMRI data were acquired using a single-shot gradient-echo (GE) echo planar imaging (EPI) pulse sequence (echo time/repetition time, 15/1500; receiver bandwidth, 250 kHz; FA, 60; FOV, 20/10; image size, 100/50; in-plane resolution, 0.2/0.2/0.8 mm; slices, 7; slice gap, 0.1). In addition, we acquired a T2-weighted 3D TurboRARE for image registration (echo time/repetition time, 21.86/2500; FOV, 20/10; image size, 180/120; in-plane resolution, 0.111/0.083/0.8 mm; slices, 7; slice gap, 0.1).

#### Calcium imaging parameters

During BOLD fMRI, we simultaneously measured the Ca^2+^ activity of a cell population in the barrel cortex. We acquired frames of 1,280 × 960 pixels at 12 bit with a frame rate of 10 Hz using custom C++ code. To acquire the Ca^2+^ imaging data, we used laser intensities between 0.1 and 0.3 mW, depending on the strength of the GCaMP6f expression. This is well below the recommended levels to prevent possible unwanted vascular modulation (<16 mW)^29^. We streamed all recorded data directly to the hard-drive of a desktop computer and synchronized the data with the recorded stimulus times.

### Data processing

#### Functional and structural MRI data preprocessing

We collected and stored all MRI data in the proprietary Bruker ParaVision format and consequently converted to BIDS through the Bruker-to-BIDS repositioning pipeline of the SAMRI package^73^. After converting the data to BIDS format, we visually assessed the alignment of the MRI-compatible microscope with the SSp-bfd region in the structural MRI scans. In the T2-weighted 3D TurboRARE images, the D_2_O-filled implant (D-content 99.9%; Sigma–Aldrich) was identifiable by a slight signal increase, while a localized signal void indicated the position of the microscope. Across all MRI scans, the microscope was consistently well aligned with the SSp-bfd. However, the spatial resolution of the MRI images was insufficient to precisely identify the voxels corresponding to the imaged region under the microscope.

Next, we used the SAMRI Generic registration workflow to preprocess the data (Supplementary Video 1)^73,74^. In this pipeline, we preprocessed the data by performing motion and slice timing corrections^75^, bias field correction^76^, registration to an atlas^73,77^ and spatiotemporal smoothening (spatial smoothing in coronal plane up to 200 µm; low-pass filtered in the temporal domain with a period threshold of 100 s).

#### Generalized linear model and time series extraction for BOLD fMRI data

We modeled volumetric data using FSL (version 6.0.4)^78^, through subject-level regression. We used six motion parameters and their first derivatives as covariates in the model. We used subject-level contrasts and variance estimates as input into the mixed-effects group level analysis using a Gaussian hemodynamic response function (phase, 0 s; sigma, 2.8 s; peak lag, 5 s). We extracted the average time series over all significantly activated voxels after cluster correction (*z*-score ≥ 3.1, FWE *P* ≤ 0.00001) and re-expressed in units of relative change, given by ΔBOLD(*t*) = (BOLD(*t*) - BOLD_0_) / BOLD_0_, where BOLD_0_ is the median filtered value (window size, 180 s) at each timestep. Owing to the difference in the temporal sampling rate between Ca^2+^ imaging and BOLD fMRI, we used linear interpolation to increase the data points of the ∆BOLD signal to match the 100-ms acquisition interval of the ∆*F*/*F* Ca^2+^ activity^79^.

#### Calcium imaging preprocessing

We implemented the following procedure to preprocess the video of each animal (Supplementary Video 2). First, we manually determined a region of interest containing fluorescence and spatially downsampled all frames by a factor of two. Next, frames that did not contain a fluorescent signal as a result of excitation laser deactivation between stimulus presentations were removed, and the TurboReg image registration algorithm^80^ was used to account for spatial translations by aligning each frame to a reference frame^79^.

#### Cell extraction and validation

We extracted cellular activity and neuropil traces using the CNMF-E pipeline^28^, using the following parameters: gSig, (5,5); gSiz, (17,17); ring_radius, 2; min_corr, 0.85; min_pnr, 8; deconvolution, constrained FOOPSI algorithm; decay_time, 0.4 (Supplementary Video 2). We evaluated the spatial and temporal components of every extracted unit on the basis of neuron shape, stability of the neural trace, temporal evolution of identified events (Ca^2+^ indicator dynamics) and the consistency of the identified events over the session, and outliers (obvious deviations from the normal distribution) were discarded. We extracted a total of 236 layer 2/3 SSp-bfd neurons (47 ± 14 per animal; mean ± s.d.; *n* = 5) from the MRI-compatible microscope recordings. We could not extract a sufficient number of cells from four animals, leading us to exclude them from analyses involving neural activity^79^.

#### Vascular activity derived from microscopy

Vascular activity was extracted from the motion-corrected microscopy videos using the multi-step vessel enhancement and profiling pipeline. For each subject, we first computed the time-averaged image of the motion-corrected stack and applied a Gaussian filter (*σ* = 50) to estimate the low-frequency background. This background was used to normalize the image, after which a region of interest (ROI) containing prominent vessels was manually selected. We then applied a two-dimensional (2D) Frangi vesselness filter to enhance vascular structures in the ROI for each frame of the video. Finally, a line profile was drawn across a target blood vessel, and the cross-sectional intensity was extracted for each frame. These profiles were concatenated into a time-series matrix representing dynamic vascular activity across the vessel diameter^79^.

#### Annotation of the vascular structure and neuron-vascular proximity

To estimate the proximity of neurons to the vasculature, we manually annotated the blood-vessel patterns for each animal (Extended Data Fig. 6). We used the time-averaged and band-pass filter (100–150 Hz) microscopic field to gain clear visibility of the vascular structure. Special care was taken to exclude features that were not part of the blood-vessel formation such as lens impurities and debris. All annotations were validated by two independent researchers.

Next, we estimated the Euclidean distance between the center of each cell and the vascular structure using the following formula:

$$d\left(p,q\right)=\,\sqrt{\mathop{\sum }\limits_{i=1}^{n}{\left({q}_{i}-{p}_{i}\right)}^{2}}\,$$

where *p* and *q* are two points in Euclidean *n* space. This description of the Euclidean distance is restricted to a 2D field due to the limited capacity of single-photon microscopy to resolve depth. Finally, we used the minimal distance value for each individual neuron to determine the neuron-vascular proximity metric.

#### Support vector machines and recursive feature elimination

To quantify the predictive information of both imaging modalities with respect to the other modality, we used a decoding approach on the basis of linear epsilon-insensitive SVM regression models. For each model, the decoding performance was quantified by the root mean square error (RMSE) according to the following equation:

$${\mathrm{RMSE}}=\,\sqrt{\frac{{\sum }_{i=1}^{N}{\left({x}_{i}-\,{\hat{x}}_{i}\right)}^{2}}{N}}$$

where *x*_i_ is the observed ∆BOLD fMRI time series and *x̂*_*i*_ the estimated ∆BOLD fMRI time series. Subsequently, the RMSE was normalized (NRMSE) by the standard deviation (*σ*_*x*_) to compare error levels between animals:

$${\mathrm{NRMSE}}\,=\,\frac{{\mathrm{RMSE}}}{{\sigma }_{x}}$$

To estimate the baseline model, we repeated this procedure with temporally permuted training features, providing an upper bound on the possible NRMSE given the prediction features.

In addition, we used recursive feature elimination to rank the predictive features on the basis of their level of contribution in predicting the alternate imaging modality. SVM-RFE uses the weight magnitude as a ranking criteria to remove less-important features recursively^30,31^, providing a subset of features that achieve the highest performance. First, an SVM regression model is trained using all available features. The resulting *β*-weights are then ranked on the basis of a predefined criterion. The feature with the lowest ranking is eliminated and given the lowest remaining rank. The process is then repeated without the eliminated feature until all features have received a ranking.

In the current paper, we used three ranking criteria, yielding three distinct rankings. (1) In the lowest absolute decoder-weight elimination, weights are ranked on the basis of the absolute *β*-weights, and the lowest value is iteratively removed. This procedure does not take into account the sign of the assigned *β*-weights, providing both positive and negative *β*-weights an equal chance at receiving a high ranking. (2) In lowest decoder-weight elimination, weights are ranked on the basis of the magnitude of the *β*-weights, and the lowest value is iteratively removed. This procedure results in a higher chance for *β*-weights with a positive value to receive a high ranking. (3) In the highest decoder-weight elimination, weights are ranked on the basis of the magnitude of the *β*-weights, and the highest value is iteratively removed. This procedure results in a higher chance for *β*-weights with a negative value to receive a high ranking.

### Software and statistical tests

For all data analysis (image preprocessing and population analysis) and statistics we used custom MATLAB and Python code. The sample sizes required for this study were initially estimated on the basis of pilot behavior studies, but we did not use formal statistical tests to predetermine sample size.

### Validation of imaging methodology

#### Perfusion

After completion of the experiments, we administered terminal anesthesia using Pentobarbital (Esconarkon; Streuli, 200 mg kg^−1^) and perfused transcardially with PBS followed by 4% paraformaldehyde (PFA). We removed brain tissue and post-fixed it for 24-48 h in 4% PFA. We then prepared coronal slices (50 μm thick) on a vibratome (VT1000 S; Leica) and stored these in PBS.

#### Verification of cell type

We used standard immunofluorescence protocols to stain inhibitory and excitatory neurons. We incubated slides overnight with either the primary antibody rabbit anti-neurogranin (1:2,000, 07-425 Millipore) or rabbit anti-GAD65 (1:500, AB1511, Millipore) at 4 °C, followed by a 2-h incubation at room temperature with the secondary antibody Alexa-Fluor-594-conjugated anti-rabbit IgG (1:200, A-11062 Invitrogen). We further stained slides for 4 min with DAPI (1:1,000, D1306, Invitrogen) in PB (0.1 M) before mounting. We then acquired confocal fluorescence images in the red (594 nm, Neurogranin or GAD65), green (488 nm, GCaMP6f) and blue (390 nm, DAPI) channels. Finally, we evaluated the resulting images with respect to their labeling overlap (Extended Data Fig. 2).

### Reporting summary

Further information on research design is available in the Nature Portfolio Reporting Summary linked to this article.

## Online content

Any methods, additional references, Nature Portfolio reporting summaries, source data, extended data, supplementary information, acknowledgements, peer review information; details of author contributions and competing interests; and statements of data and code availability are available at 10.1038/s41592-026-03154-2.

## Supplementary information

Reporting Summary

Supplementary Video 1 Motion correction of Calcium imaging and BOLD fMRI recordings. The video demonstrates the application of motion correction to simultaneously acquired BOLD fMRI and calcium imaging data. The upper left panel shows the raw BOLD fMRI signal prior to preprocessing. The upper right panel displays the corresponding data after preprocessing, registration, and motion correction. The lower left panel presents the raw calcium imaging recordings, and the lower right panel illustrates the same data following motion correction. The comparison highlights the reduction of motion-related artifacts and the improved spatial stability of the recordings.

Supplementary Video 2 Calcium imaging raw data and ΔF/F. The video shows raw calcium imaging recordings (left) and the corresponding ΔF/F signal (right), obtained by normalizing fluorescence changes to the baseline intensity to correct for slow drifts and enable comparison across time and regions.

## Figures and Tables

**Extended Data Table 1:**
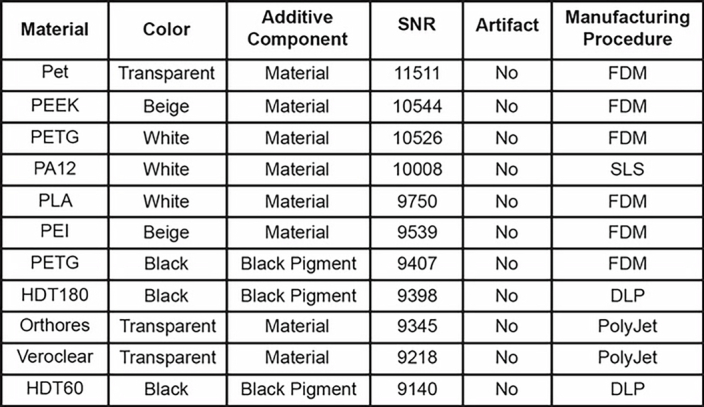
BOLD fMRI SNR

Table showing the BOLD fMRI signal to noise (SNR) values of tested materials. Higher SNR values correspond to better quality BOLD fMRI images.

## Data Availability

Source data is publicly available via figshare (10.6084/m9.figshare.31389115.v1). Due to substantial size and complexity, all other data (raw imaging data and pre-processed activity data) that support the findings of this study will be made available upon request.

## Code availability

The MATLAB and Python code scripts detailing all aspects of the performed analysis are made publicly available on github (https://github.com/rlemubaghs/camri).
