# Supplementary material for: Simultaneous single-cell calcium imaging of neuronal population activity and brain-wide BOLD fMRI
Source: Nat Methods. 2026 Jul 15;23(8):1637–46. doi: 10.1038/s41592-026-03154-2 (PMC13441971; doi:10.1038/s41592-026-03154-2)
Supplement: Supplementary file 1 [file 41592_2026_3154_MOESM1_ESM.pdf]

nature portfolio

Corresponding author(s): Benjamin F. Grewe

Last updated by author(s): Nov 2, 2023

Reporting Summary

Nature Portfolio wishes to improve the reproducibility of the work that we publish. This form provides structure for consistency and transparency in reporting. For further information on Nature Portfolio policies, see our Editorial Policies and the Editorial Policy Checklist.

Please do not complete any field with "not applicable" or n/a. Refer to the help text for what text to use if an item is not relevant to your study.
For final submission: please carefully check your responses for accuracy; you will not be able to make changes later.

Statistics

For all statistical analyses, confirm that the following items are present in the figure legend, table legend, main text, or Methods section.

n/a	Confirmed	
☐	☒	The exact sample size (*n*) for each experimental group/condition, given as a discrete number and unit of measurement
☐	☒	A statement on whether measurements were taken from distinct samples or whether the same sample was measured repeatedly
☐	☒	The statistical test(s) used AND whether they are one- or two-sided<br>*Only common tests should be described solely by name; describe more complex techniques in the Methods section.*
☐	☒	A description of all covariates tested
☐	☒	A description of any assumptions or corrections, such as tests of normality and adjustment for multiple comparisons
☐	☒	A full description of the statistical parameters including central tendency (e.g. means) or other basic estimates (e.g. regression coefficient) AND variation (e.g. standard deviation) or associated estimates of uncertainty (e.g. confidence intervals)
☐	☒	For null hypothesis testing, the test statistic (e.g. *F*, *t*, *r*) with confidence intervals, effect sizes, degrees of freedom and *P* value noted<br>*Give P values as exact values whenever suitable.*
☒	☐	For Bayesian analysis, information on the choice of priors and Markov chain Monte Carlo settings
☒	☐	For hierarchical and complex designs, identification of the appropriate level for tests and full reporting of outcomes
☒	☐	Estimates of effect sizes (e.g. Cohen's *d*, Pearson's *r*), indicating how they were calculated

Our web collection on statistics for biologists contains articles on many of the points above.

Software and code

Policy information about availability of computer code

Data collection	All in vivo calcium imaging data was collected with a custom MR compatible microscope.<br>All in vivo BOLD MRI data was collected with a Bruker Biospin 7T/11 cm horizontal bore scanner (BioSpec 70/20 USR) fitted with a RF RES 300 1H 089/072 QSN TR AD volume coil combined and an RF SUC 300 1H LNA AV3 surface coil (BRUKER BioSpin MRI GmbH).<br>All histological images were acquired using a either CLSM - Leica Stellaris 5 upright microscope equipped with an HC PL APO CS2 20x 0.75 IMM 0.66 objective(data acquisition software used was LAS X) or an Olympus fluorescence microscope (BX51).<br>All data was collected using custom MATLAB and LabVIEW code.
Data analysis	We used MATLAB and Python for the calcium imaging data preprocessing, motion correction, and cell extraction. For the MRI data preprocessing, motion correction and signal extraction we used FSL.<br>For all combined calcium imaging and BOLD fMRI data analysis we developed custom code in the MATLAB programming environment.

For manuscripts utilizing custom algorithms or software that are central to the research but not yet described in published literature, software must be made available to editors and reviewers. We strongly encourage code deposition in a community repository (e.g. GitHub). See the Nature Portfolio guidelines for submitting code & software for further information.

Data

Policy information about availability of data

All manuscripts must include a data availability statement. This statement should provide the following information, where applicable:

- Accession codes, unique identifiers, or web links for publicly available datasets
- A description of any restrictions on data availability
- For clinical datasets or third party data, please ensure that the statement adheres to our policy

The source data that support the findings of this study will be included in the paper. The raw imaging data will be made available by the corresponding author upon reasonable request. Figure source data will be accessible in Figshare (link will be added upon acceptance).

Human research participants

Policy information about studies involving human research participants and Sex and Gender in Research.

Reporting on sex and gender	N/A
Population characteristics	N/A
Recruitment	N/A
Ethics oversight	N/A

Note that full information on the approval of the study protocol must also be provided in the manuscript.

Field-specific reporting

Please select the one below that is the best fit for your research. If you are not sure, read the appropriate sections before making your selection.

☒ Life sciences ☐ Behavioural & social sciences ☐ Ecological, evolutionary & environmental sciences

For a reference copy of the document with all sections, see nature.com/documents/nr-reporting-summary-flat.pdf

Life sciences study design

All studies must disclose on these points even when the disclosure is negative.

Sample size	We used sample sizes comparable to other papers that measured multiple modalities (Lake et al., 2021: N = 6)
Data exclusions	We did not initiate data collection in three animals because they did not reach the specified motion criteria (average FWD ≤ 0.05 mm, and at least 50% of trials showed no excessive motion defined as FWD spikes > 0.075 mm).<br>In four animals we did not observe any single-cell neuronal activity, due to insufficient labeling and/or cortical window misplacement. These animals did demonstrate high quality for the BOLD fMRI and neuropil data, and were included for analysis that only looked at this data type.
Replication	We replicate the analysis for all findings multiple times to ensure findings were not based on chance.
Randomization	Only one stimulus was used. Data was collected by both R.U. and R.B. to minimize experimenter influence. Female cohort was added on the request from reviewers after male data collection was finished.
Blinding	R.U. and R.B. performed all animal experiments. Since animals need to be prepared and handled individually and due to the nature of the experiment a blinding of the experimenter was not possible.

Reporting for specific materials, systems and methods

We require information from authors about some types of materials, experimental systems and methods used in many studies. Here, indicate whether each material, system or method listed is relevant to your study. If you are not sure if a list item applies to your research, read the appropriate section before selecting a response.

Materials & experimental systems

n/a	Involved in the study
☐	☒ Antibodies
☒	☐ Eukaryotic cell lines
☒	☐ Palaeontology and archaeology
☐	☒ Animals and other organisms
☒	☐ Clinical data
☒	☐ Dual use research of concern

Methods

n/a	Involved in the study
☒	☐ ChIP-seq
☒	☐ Flow cytometry
☐	☒ MRI-based neuroimaging

Antibodies

Antibodies used

rabbit anti-GAD65+67 (1:500, AB1511, Millipore)
rabbit anti-Neurogranin (1:2000, 07-425, Millipore)
Alexa 594 anti-rabbit (1:200, A-11062, Invitorgen)
DAPI (1:1000, D1306, Invitrogen)

Validation

Antibodies were chosen based on a literature review for each antibody to identify the best candidate for our experiments. Validation was determined by reviewing the manufacturer's literature, other published research, and prior experiments in the lab. For each experiment, at least one slide was designated for a "secondary only" control and examined for potential background staining.
Commercial antibodies were validated by the manufacturer:
rabbit anti-GAD65+67 www.merckmillipore.com/CH/de/product/Anti-Glutamate-Decarboxylase-65-67-Antibody,MM_NF-AB1511
rabbit anti-Neurogranin https://www.merckmillipore.com/CH/de/product/Anti-Neurogranin,MM_NF-07-425-I-100UG
Alexa 594 anti-rabbit www.thermofisher.com/antibody/product/Rabbit-anti-Mouse-IgG-H-L-Cross-Adsorbed-Secondary-Antibody-Polyclonal/A-11062
DAPI www.thermofisher.com/order/catalog/product/D1306

Animals and other research organisms

Policy information about studies involving animals; ARRIVE guidelines recommended for reporting animal research, and Sex and Gender in Research

Laboratory animals

Experiments were performed on Rasgrf2-2A-dCre mice (Jackson Laboratory, stock number 022864) crossbred with Ai148D mice (Jackson Laboratory, stock number 030328). All mice were males aged 4 - 6 months (25-35 g) during in vivo imaging experiments. Animals were housed in individually ventilated cages (IVC) in a 12 h light/dark cycle room (lights on from 7:00 to 7:00), and were provided food and water ad libitum. During the experiments mice were single housed.

Wild animals

N/A

Reporting on sex

We report the sex of all animals used for this study in the methods section (all male).

Field-collected samples

N/A

Ethics oversight

All animal well-fare and ethical aspects of our study were evaluated by the Swiss Cantonal Veterinary office and approved,.

Note that full information on the approval of the study protocol must also be provided in the manuscript.

Magnetic resonance imaging

Experimental design

Design type

We use a block design

Design specifications

We presented each mouse with 12 trials (block design) consisting of 10 seconds pneumonic (air) stimulation at 3 Hz (40 ms duty cycle) to the full whisker pad located contralaterally to the MRI compatible microscope.

Behavioral performance measures

The mice were passively experiencing whisker stimulation and did not need to perform a specific task.

Acquisition

Imaging type(s)

Functional and structural MRI

Field strength

7T

Sequence & imaging parameters

BOLD fMRI data was acquired using a single-shot gradient-echo (GE) echo planar imaging (EPI) pulse sequence (TE/TR:

Sequence & imaging parameters	15/1500, BW: 250 kHz, FA: 60, FOV: 20/10, Image Size: 100/50, In Plane resolution: 0.2/0.2/0.8 mm, Slices: 7, Slice Gap: 0.1). Structural scans were acquired using a T2-weighted 3D TurboRARE (TE/TR: 21.86/2500, FOV: 20/10, Image Size: 180/120, we acquired the in-plane resolution: 0.111/0.083/0.8 mm, Slices: 7, Slice Gap: 0.1) for image registration.
Area of acquisition	7 slices were collected during each scan covering the whole brain except Cerebellum and Olfactory-bulb.

Diffusion MRI ☐ Used ☒ Not used

Preprocessing

Preprocessing software	We used the Generic registration workflow of the SAMRI package to preprocess the data (Ioanas et al. 2021, 2020). The data was preprocessed by performing motion and slice timing corrections, bias field correction, registration to an atlas, and spatiotemporal smoothening (spatial smoothing in coronal plane up to 200 µm; low-pass filtered in the temporal domain with a period threshold of 100 seconds).
Normalization	Data was normalized to the template (Dorr et al., 2008). First, the structural image was aligned with the atlas. The parameters acquired through this alignment were then used to transform the motion corrected functional images.
Normalization template	We used the template described in Dorr et al. (2008). The atlas consists of a high resolution three-dimensional brain scan that was created by averaging the magnetic resonance image of 40 adult C57Bl/6J mice.
Noise and artifact removal	We used all six motion parameters (extracted during motion correction using SAMRI) and their first derivatives as covariates in FSL (version 6.0.4) during subject level regression.
Volume censoring	No volume was censored

Statistical modeling & inference

Model type and settings	We modeled the acquired volumetric data using FSL (version 6.0.4), through subject level regression. We used six motion parameters and their first derivatives as covariates in the model. We used subject level contrasts and variance estimates as input into the mixed effects group level analysis using a gaussian hemodynamic response function (phase = 0s, sigma = 2.8s, peak lag = 5s).
Effect(s) tested	We compared the stimulus on condition (whisker stimulation to the whisker bedding contralateral to the microscope) with the baseline condition (stimulus off).

Specify type of analysis: ☐ Whole brain ☐ ROI-based ☒ Both

Anatomical location(s)	The anatomical locations were based on the template atlas described in Dorr et al. (2008).
Statistic type for inference (See Eklund et al. 2016)	Voxel-wise
Correction	We used FWE with a P value of ≤ 0.00001

Models & analysis

n/a	Involved in the study
☒	☐ Functional and/or effective connectivity
☒	☐ Graph analysis
☐	☒ Multivariate modeling or predictive analysis

Multivariate modeling and predictive analysis	We used a linear epsilon-insensitive SVM regression models to predict between both modalities (from BOLD fMRI to calcium imaging and vice versa). For each model, the decoding performance was quantified by the Root Mean Square Error (RMSE). To estimate the baseline model, we repeated this procedure with temporally permuted training features, providing an upper bound on the possible NRMSE given the prediction features.<br>We used this setting in two analysis: (1) to predict the BOLD fMRI signal in the SSp-bfd from the neural activity at the single cell level, and (2) to predict the average neural activity from cells in the SSp-bfd from the BOLD fMRI activity in different areas across the brain.<br>In the second analysis we applied PCA to the BOLD fMRI data to decrease the number of input dimensions (20 components explained ≥ 75% of the variance) to avoid overfitting due to the high number of significantly activated voxels.

This checklist template is licensed under a Creative Commons Attribution 4.0 International License, which permits use, sharing, adaptation, distribution and reproduction in any medium or format, as long as you give appropriate credit to the original author(s) and the source, provide a link to the Creative Commons license, and indicate if changes were made. The images or other third party material in this article are included in the article's Creative Commons license, unless indicated otherwise in a credit line to the material. If material is not included in the article's Creative Commons license and your intended use is not permitted by statutory regulation or exceeds the permitted use, you will need to obtain permission directly from the copyright holder. To view a copy of this license, visit http://creativecommons.org/licenses/by/4.0/
